# Three smart spectrophotometric methods for resolution of severely overlapped binary mixture of Ibuprofen and Paracetamol in pharmaceutical dosage form

**DOI:** 10.1186/s13065-019-0618-3

**Published:** 2019-08-06

**Authors:** Christine M. El-Maraghy, Nesrine T. Lamie

**Affiliations:** 1 Analytical Chemistry Department, Faculty of Pharmacy, October University for Modern Sciences and Arts (MSA), 6th October City, 11787 Egypt; 2 Pharmaceutical Chemistry Department, Faculty of Pharmacy, King Abdulaziz University, Jeddah, Saudi Arabia; 3 Analytical Chemistry Department, Faculty of Pharmacy, Cairo University, Giza, Egypt

**Keywords:** Ibuprofen, Paracetamol, Ratio difference, Constant center, Mean centering, Spectrophotometry

## Abstract

Paracetamol is an analgesic-antipyretic drug and Ibuprofen is a non-steroidal anti-inflammatory drug. They are co-formulated as tablets to improve analgesia, to simplify prescribing and to improve patient compliance. Three accurate, simple and sensitive spectrophotometric methods were developed for the simultaneous determination of Paracetamol and Ibuprofen in their co-formulated dosage form. The first method was the ratio difference, which was based on the measurement of the difference in absorbance between the two wavelengths (210.6 and 216.4 nm) for Ibuprofen and (236.0 and 248.0 nm) for Paracetamol. The second method was constant center method which depends on using the constant found in the ratio spectra. The third method was the mean centering of ratio spectra which measured the manipulated values at 240 nm and 237 nm for Ibuprofen and Paracetamol, respectively. Beer’s law was obeyed in the concentration range of 2–50 μg/mL for Ibuprofen and 2–20 μg/mL for Paracetamol. The recovery % of the accuracy of both methods ranged from 99.64 to 100.56%. Factors affecting the resolution of the spectra were studied and optimized. The three methods are validated according to ICH guidelines and could be applied for the pharmaceutical preparation.

## Introduction

**Fig. 1:**
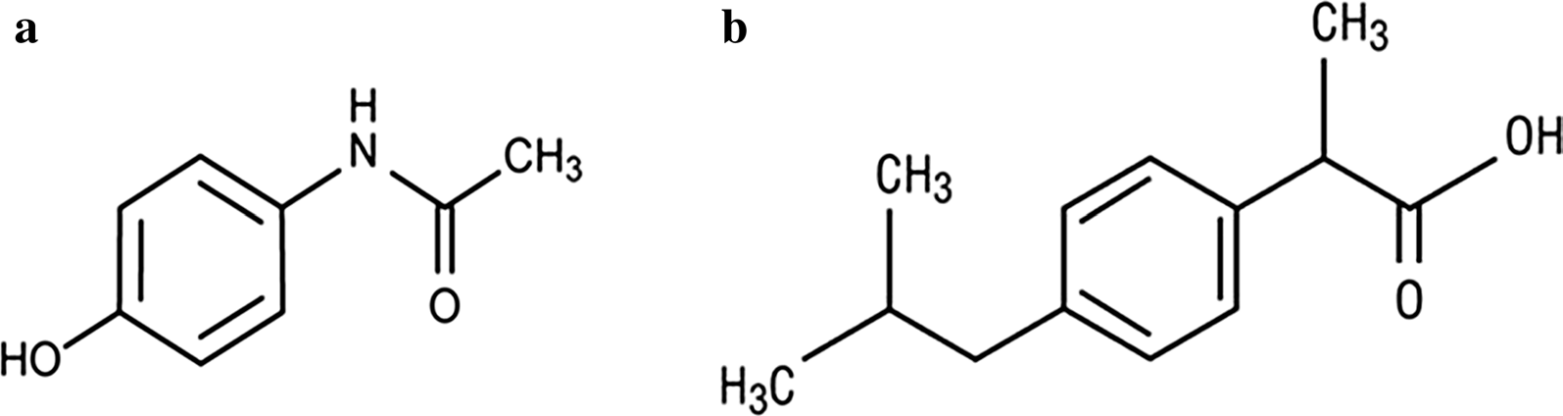
Chemical structures of **a** Paracetamol (PAR) and **b** Ibuprofen (IBU)

Paracetamol (PAR); *N*-acetyl-*p*-aminophenol (Fig. 1a), is an effective alternative to aspirin as an analgesic–antipyretic agent but its anti-inflammatory effect is much weaker than Aspirin [1]. Ibuprofen (IBU) (Fig. 1b), is the first member of the propionic acid class of non-steroidal anti-inflammatory drugs, it used in the symptomatic treatment of rheumatoid arthritis, osteoarthritis and as analgesic [1]. The two drugs have been co-formulated to improve analgesia compared with their single-dose administration, to simplify prescribing and to improve patient compliance [2]. The literature review reveals the determination of this binary mixture of PAR and IBU using spectrophotometric methods such as simultaneous equation and absorbance ratio methods [3], derivative methods [4, 5] and chemometric-assisted spectrophotometry [6]. Fourier transform infrared spectroscopy [7], spectrofluorimetry [8] and HPLC methods [9–13] were also reported. There are three published spectrophotometric methods for their simultaneous determination but they used manipulations and derivatization which depends on one wavelength for amplitude measurement which may cause an error with the small absorbance values. The aim of our work was to develop more simple and sensitive spectrophotometric methods than the published one for the resolution of severely overlapped spectra of PAR and IBU and their determination in tablet dosage from without interference from the excipients. The three developed methods are simpler (they involved fewer data processing steps) and more accurate than the previously published spectrophotometric methods as they did not use the derivatization or the multiple manipulating steps; so the signal-to-noise ratio was improved.

## Experimental

### Apparatus

Shimadzu UV1800 double beam UV/Visible spectrophotometer (Japan) with 1 cm quartz cells. Matlab^®^ (8.3.0.532) R2014a software (The Mathworks, Natick, USA) with PLS toolbox 2.1 was used for the mean centering spectrophotometric method calculation.

### Pure standards

IBU standard was obtained as a kind gift sample from Unipharma Company, Cairo, Egypt. PAR standard was obtained from SIGMA pharmaceutical industries, Cairo, Egypt. Standard IBU and PAR were with claimed purity of 99.63%, and 100.25%; respectively as per the reported spectrophotometric method [5].

### Chemicals and reagents

Methanol was obtained from Carlo Erba Reagents, Italy.

### Pharmaceutical formulations

Cetafen^®^ tablets (Batch No. 51115) labeled to contain 200 mg IBU and 325 mg PAR manufactured by SIGMA pharmaceutical industries, Egypt. Parofen^®^ tablets (Batch No. 9472) labeled to contain 400 mg IBU and 500 mg PAR manufactured by Unipharma company, Egypt.

### Preparation of standard solutions

IBU and PAR stock solutions of 1 mg/mL were prepared in methanol. The working standard solutions of each drug were prepared by dilution from the stock solution with methanol of concentration (100 μg/mL).

### Laboratory prepared mixtures

Solutions of different concentrations of IBU and PAR were prepared by transferring aliquots from the corresponding working solutions into 10-mL volumetric flasks and the volume was completed with methanol.

## Procedures

### Linearity and construction of calibration curves

#### Ratio difference spectrophotometric method (RD)

Aliquots equivalent to (0.2–5 mL) were transferred from the working standard solutions of PAR and IBU into a series of 10–mL volumetric flasks, and the volume was completed with methanol to obtain a concentration of (2–20 μg/mL) for PAR and (2–50 μg/mL) for IBU. The zero order absorption spectra of the prepared solutions were measured over the range 200–400 nm. The spectra of PAR prepared solutions were divided by the spectrum of 5 μg/mL IBU and the spectra of IBU solutions were divided by the spectrum of 8 μg/mL PAR. The difference in peak amplitudes between the two selected wavelengths 236 and 248 nm for PAR and 210.6 and 216.4 nm for IBU were calculated. Calibration graphs relating the differences in the peak amplitudes at the chosen wavelength versus the corresponding concentrations were constructed.

#### For constant center method (CC)

Using the same previous prepared series of concentration (2–20 μg/mL) for PAR and (2–50 μg/mL) for IBU. The spectra of PAR prepared solutions were divided by the spectrum of 5 μg/mL IBU (divisor) and the spectra of IBU solutions were divided by the spectrum of 8 μg/mL PAR. The difference in amplitudes of the obtained ratio spectra between the two selected wavelengths 236 and 248 nm versus amplitudes of ratio spectra at 236 nm for IBU and 210.6 and 216.4 nm versus amplitudes of ratio spectra at 216.4 nm for PAR were calculated and the regression equations were computed.

#### Mean centering of ratio spectra method (MCR)

The previous scanned spectra for both drugs are exported to Matlab^®^software. The spectra of IBU prepared solutions were divided by spectrum of PAR (8 μg/mL) and the obtained ratio spectra were mean centered. In the same way, the spectra of PAR solutions were divided by the spectrum of IBU (5 μg/mL) and the obtained ratio spectra were mean centered. The calibration curves were constructed by plotting the mean centered values at 240 nm and 237 nm, for IBU and PAR, respectively versus their corresponding concentrations.

#### Analysis of laboratory prepared mixtures

The three described methods were applied to laboratory prepared mixtures containing different concentration of PAR and IBU. The recovery % of PAR and IBU were calculated.

#### Application to pharmaceutical preparations

For Parofen^®^ tablets; ten tablets were finely powdered. An amount of the powdered tablets equivalent to 0.96 g was accurately weighted and transferred into 100-mL beaker; dissolved in about 60 mL methanol, the mixture was sonicated for 15 min then filtered into 100-mL volumetric flask and the volume was completed with methanol. Then 5.0 mL from this stock solution was diluted into 100-mL volumetric flask and completed to the mark with methanol (IBU 0.2 mg/mL and PAR 0.25 mg/mL). A dilution was prepared by transferring 1 mL from this working solution into 50-mL volumetric flask and completed with methanol (IBU 4 μg/mL and PAR 5 μg/mL). The same procedure was applied for Cetafen^®^ tablets to prepare a solution of concentration (IBU 10 μg/mL and PAR 16.25 μg/mL). The proposed procedures were applied to determine the concentration of each drug in the pharmaceutical preparations.

## Results and discussion

**Fig. 2:**
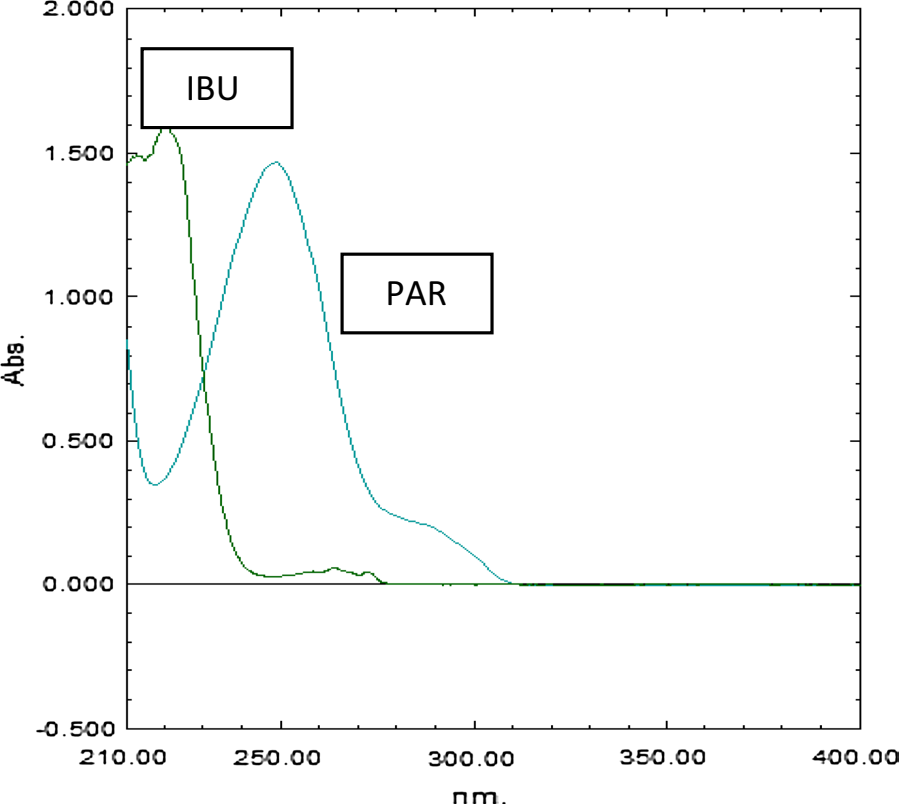
Zero order overlay absorption spectra of IBU (40 μg/mL) and PAR (16 μg/mL)

The aim of this work was to develop simple, sensitive and validated spectrophotometric methods for simultaneous determination of IBU and PAR in their pharmaceutical preparations without pre-separation step to be applied in the quality control labs. The three proposed methods were compared to the previously published spectrophotometric methods [3–5]. They are found to be simpler and more sensitive as they did not use derivative or multiple manipulating steps. The zero order absorbance spectra of IBU and PAR in methanol displayed an overlap (Fig. 2), so the direct UV cannot be used for their simultaneous analysis.

### Ratio difference spectrophotometric method (RD)

**Fig. 3:**
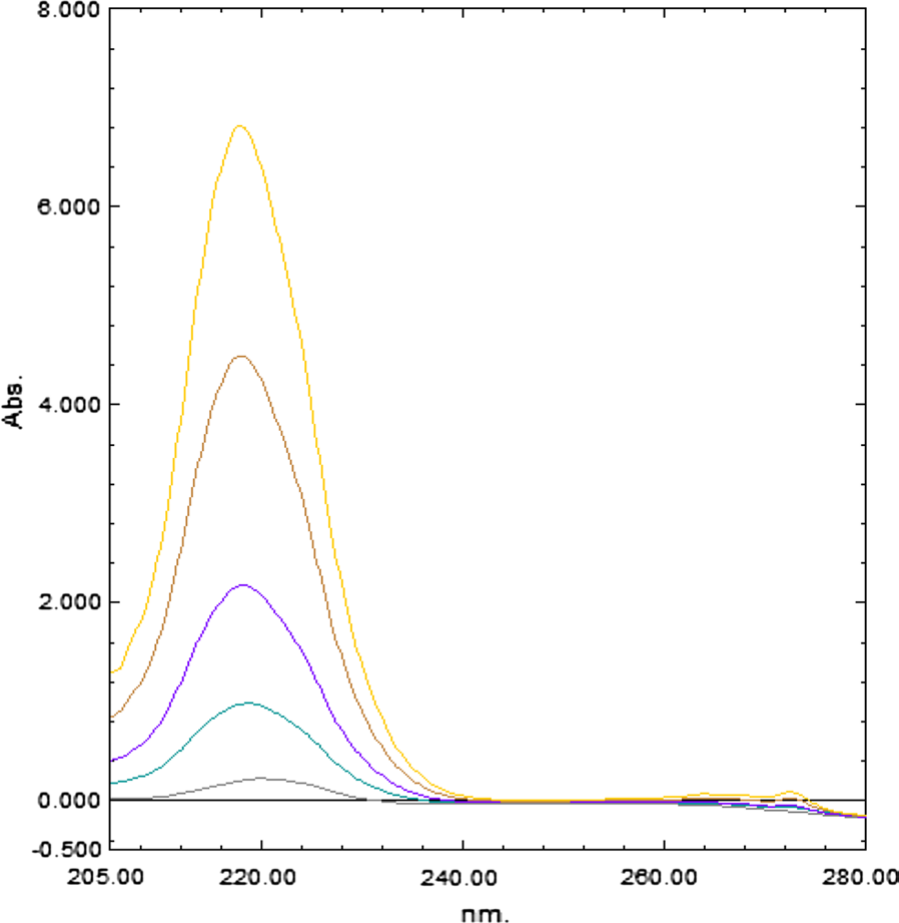
Ratio spectra of different concentration of IBU (2–50 μg/mL), using 8 μg/mL of PAR as divisor

**Fig. 4:**
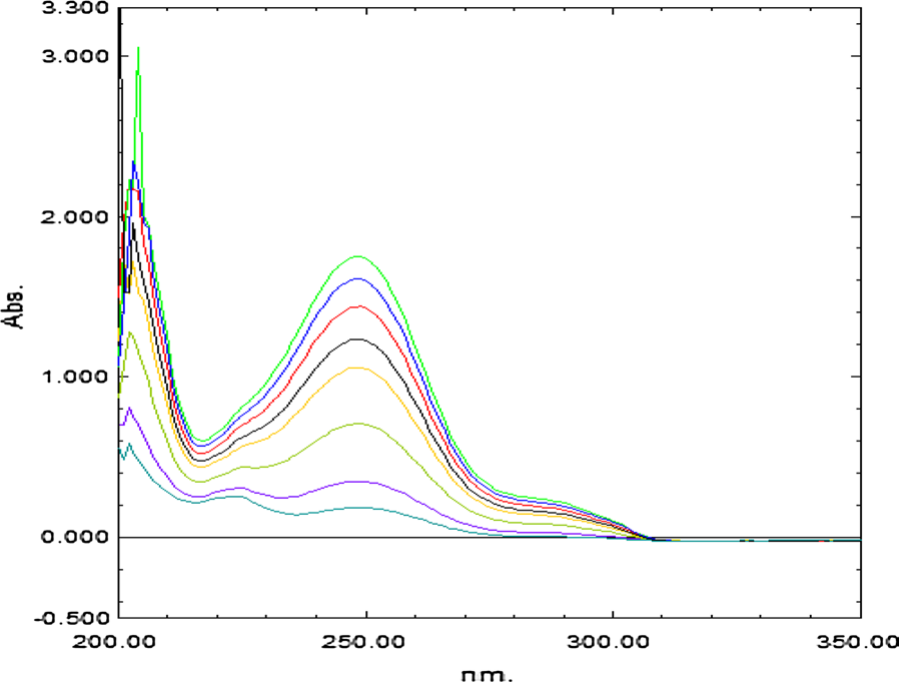
Ratio spectra of different concentration of PAR (2–20 μg/mL), using 5 μg/mL of IBU as divisor

The main characteristics of this method are its simplicity of calculations, rapidity and accuracy. The two main significant factors are the choice of the divisor and the selection of the two wavelengths [14–18]. Different wavelengths ratio were tried to obtain the best linearity. Different divisor concentrations were tried in order to give minimal noise and maximum sensitivity. The divisor concentrations of 8 μg/mL PAR and 5 μg/mL IBU gave the best results (Figs. 3, 4). The advantage of this method over the previously published methods was that it did not need critical measurement at one fixed wavelength hence signal to noise ratio was enhanced.

### Constant center method (CC)

This recently developed method [19, 20] depends on using the constants present in the ratio spectra which could be manipulated to obtain the zero order spectra of the two analytes in mixture and enable to measure them at their λ_max_, which offers maximum accuracy and precision with minimum manipulation steps. For the determination of IBU in the binary mixture; the ratio spectra of the binary mixtures obtained by using 5 μg/mL IBU′ as a divisor represents {(PAR/IBU′) + constant}, than the ratio difference at two selected wavelength {236 nm (λ_1_) and 248 nm (λ_2_)} was calculated {(PAR/IBU′)^1^ + (PAR/IBU′)^2^}, so the analyte (IBU) was cancelled. The ratio amplitude of the mixtures at 236 nm were recorded {(PAR/IBU′) + (IBU/IBU′)} for each mixture, while the postulated ratio amplitude value of (PAR/IBU′) can be calculated by using the regression equation representing the direct relationship between the ratio difference of ratio spectra at 236 nm and 248 nm versus the corresponding ratio amplitudes at 236 nm.

$${\text{P}}_{ 2} - {\text{P}}_{ 1} = 0.3916 {\text{ P}}_{ 1} - 0.0114, \quad {\text{r}} = 0.9999$$

where P_1_, P_2_ are the ratio amplitudes at 236 nm and 248 nm of the ratio spectra of concentration range of PAR (2–20 µg/mL) using 5 µg/mL IBU′ as a divisor.

The constant value was calculated as follow {∆P = (P_recorded_ − P_postulated_)}, measuring the difference between the recorded amplitude and postulated amplitude at 236 nm.

$${\text{Constant value (CV)}} = \left[ {{\text{P}}_{\text{recorded}} - {\text{ P}}_{\text{postulated}} } \right],$$

where P_recorded_ is the recorded amplitude of the ratio spectra of the laboratory prepared mixtures using 5 µg/mL IBU′ as a divisor at 236 nm and P_postulated_ is the calculated amplitude using the specified regression equation.

The original spectrum of IBU in the mixture can be obtained by multiplying the obtained constant (IBU/IBU′) of the laboratory mixtures by IBU′ (the divisor), which is used for direct determination of IBU from the corresponding regression equation obtained by plotting the absorbance values of the zero order spectra at its λ 236 nm against the corresponding concentrations of IBU.

PAR can be determined by repeating the same steps using a spectrum of 8 µg/mL PAR′ as a divisor to calculate the constant value of PAR using the following regression equation.

$${\text{P}}_{ 1} - {\text{P}}_{ 2} = 0.5544 {\text{P}}_{ 1} + 0.0318, \quad {\text{r}} = 0.9999$$

where P_1_, P_2_ are the ratio amplitudes at 210.6 nm and 216.4 nm of the ratio spectra of IBU (2–50 µg/mL) using 8 µg/mL PAR′ as a divisor versus the corresponding ratio amplitudes at 216.4 nm. The original spectrum of PAR is obtained after multiplication of the calculated constant value by the 8 μg/mL PAR′.

### Mean centering of ratio spectra method (MCR)

**Fig. 5:**
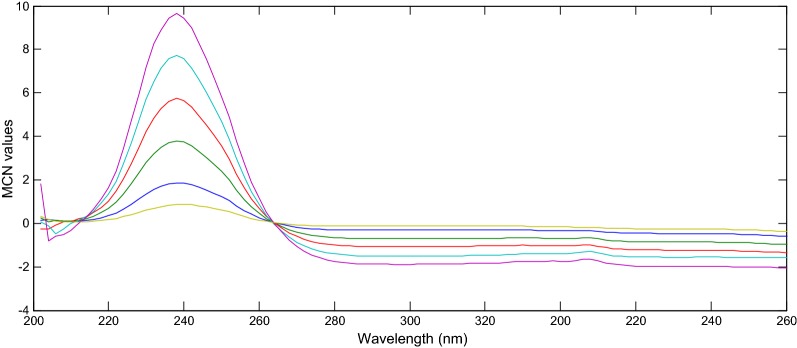
Mean centered ratio spectra of IBU (2–30 μg/mL), using 8 μg/mL of PAR as divisor at 240 nm

**Fig. 6:**
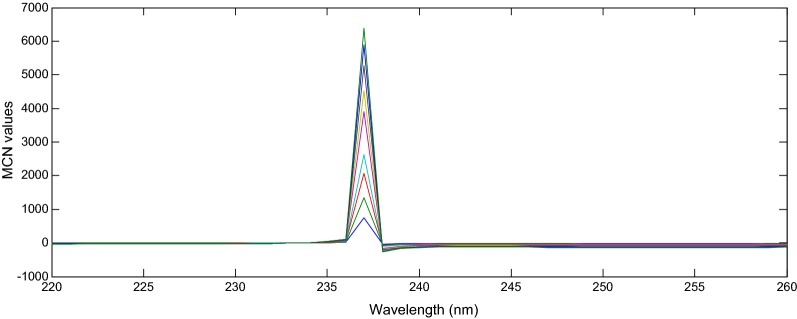
Mean centered ratio spectra of PAR (2–20 μg/mL), using 5 μg/mL of IBU as divisor at 237 nm

Mean centering method depended on the manipulation of the ratio spectra by the Matlab^®^ software to delete the effect of one component of the mixture to determine the other one, and it also eliminates the derivative step [21]. The ratio spectra of IBU and PAR were obtained using (8 µg/mL of PAR) and (5 μg/mL IBU) as divisors, respectively and were then mean centered, as shown in Figs. 5 and 6.

## Method validation

The international conference on Harmonization (ICH) guidelines [22] were followed for validation of the proposed methods. The calibration curves show a good linearity in the concentration range (2–20 μg/mL) for PAR and (2–50 μg/mL) for IBU for the two methods. Accuracy was checked by analysis of pure samples of IBU and PAR, where satisfactory results were obtained. The intra- and inter-day precision was evaluated by analysis three different concentrations of each drug in triplicate on the same day and on three successive days. The detection and quantitation limits were calculated using the approach based on the standard deviation of the response and the slope; the results are shown in Table 1. Specificity of the methods was performed by the analysis of laboratory prepared mixtures of PAR and IBU within the linearity range. Good results were shown in Table 2.

**Table 1:** Analytical parameters and validation results of the determination of PAR and IBU by the proposed methods

Parameter	Ratio difference		Constant center		Mean centering	
	PAR	IBU	PAR	IBU	PAR	IBU
Linearity (μg/mL)	2–20	2–50	2–20	2–50	2–20	2–50
Slope	0.0538	0.1267	0.5544	0.3916	318.22	0.1949
Standard error of the slope	0.00067	0.00084	0.00176	0.00107	3.770	0.000354
Intercept	0.009016	0.1203	0.0318	− 0.0114	108.66	0.5010
Standard error of intercept	0.001722	0.02372	0.01096	0.00091	50.507	0.01177
Standard deviation of residuals from line	0.01033	0.03782	0.01637	0.00109	57.851	0.01122
Accuracy (mean ± SD)	99.72 ± 1.71	100.11 ± 0.53	99.64 ± 0.803	99.97 ± 0.641	100.21 ± 1.24	100.56 ± 0.36
Intraday precision (RSD%)^a^	0.14	0.42	0.36	0.57	0.44	0.75
Interday precision (RSD%)^b^	0.57	0.45	0.78	0.64	1.21	1.04
LOD	0.63	0.985	0.097	0.01	0.59	0.189
LOQ	1.537	1.985	0.295	0.0278	1.81	0.575

^a^Intraday precision: average of 3 different concentrations in triplicate (n = 9) within the same day

^b^Interday precision:average of 3 different concentrations in triplicate (n = 9) repeated on 3 successive days

**Table 2:** Determination of the studied drugs in the laboratory prepared mixtures

Ratio PAR:IBU	Ratio difference (recovery %)^a^		Constant center		Mean centering (recovery %)^a^	
	PAR	IBU	PAR	IBU	PAR	IBU
2:1	99.44	100.74	98.65	99.87	97.87	95.16
1:2	98.42	99.39	99.34	100.95	98.54	99.87
2:2	101.07	98.87	100.50	99.53	99.93	98.63
3:2	99.41	100.10	100.74	101.08	100.14	98.41
5:4	101.03	99.65	101.22	99.54	101.67	100.26

^a^Average of three separate determinations

### Application to pharmaceutical preparations

The proposed methods were applied for the determination of PAR and IBU in pharmaceutical preparations; and the validity of the proposed procedures was confirmed by applying the standard addition technique showing no interference from excipients. The results obtained were shown in Table 3.

**Table 3:** Determination of PAR and IBU in pharmaceutical preparation and application of standard addition technique

(A) Market preparation: Cetafen^®^ tablet claimed to contain 325 mg PAR and 200 mg IBU									
Proposed method recovery %^a^						Standard addition technique			
						Ratio difference			
Ratio difference		Mean centering		Constant center		Taken (μg/mL)		Recovery %^a^	
PAR	IBU	PAR	IBU	PAR	IBU	PAR	IBU	PAR	IBU
100.12 ± 0.62	101.34 ± 1.51	99.58 ± 0.53	100.52 ± 1.26	99.68 ± 0.95	100.44 ± 1.17	3.0	20.0	100.76	99.16
						3.0	20.0	101.53	100.34
						3.0	20.0	100.42	101.95

Standard addition technique							
Mean centering				Constant center			
Taken (μg/mL)		Recovery %^a^		Taken (μg/mL)		Recovery %^a^	
PAR	IBU	PAR	IBU	PAR	IBU	PAR	IBU
3.0	20.0	98.14	100.96	3.0	20.0	100.37	99.02
3.0	20.0	100.33	100.55	3.0	20.0	98.43	100.55
3.0	20.0	101.08	101.13	3.0	20.0	100.85	99.89

(B) Market preparation: Parofen^®^ tablet claimed to contain 500 mg PAR and 400 mg IBU									
Proposed method recovery %^a^						Standard addition technique			
						Ratio difference			
Ratio difference		Mean centering		Constant center		Taken (μg/mL)		Recovery %^a^	
PAR	IBU	PAR	IBU	PAR	IBU	PAR	IBU	PAR	IBU
98.72 ± 0.89	100.74 ± 0.56	100.58 ± 0.93	101.22 ± 1.18	9936 ± 0.52	100.36 ± 1.03	3.0	20.0	100.54	99.96
						3.0	20.0	101.24	101.31
						3.0	20.0	101.43	100.63

Standard addition technique							
Mean centering				Constant center			
Taken (μg/mL)		Recovery %^a^		Taken (μg/mL)		Recovery %^a^	
PAR	IBU	PAR	IBU	PAR	IBU	PAR	IBU
3.0	20.0	100.73	100.13	3.0	20.0	101.33	99.45
3.0	20.0	101.12	99.58	3.0	20.0	100.95	99.82
3.0	20.0	100.37	100.22	3.0	20.0	100.04	98.73

^a^Average of three separate determinations

### Statistical comparison

PAR and IBU binary mixture was determined previously by different spectrophotometric methods. The proposed ratio difference method is simpler and more accurate than the previously published derivative and derivative ratio methods [4, 5] as there are no derivative steps therefore signal-to-noise ratio was enhanced. It is also simpler than simultaneous equation method and absorbance ratio method [3] as they involve several tedious mathematical calculations. Table 4 showed statistical comparisons of the results obtained by the proposed methods and the reported spectrophotometric method [5]. The calculated *t* and F values were less than the theoretical ones indicating that there was no significant difference between the reported and the proposed method regarding the accuracy and precision.

**Table 4:** Statistical comparison for the results obtained by the proposed spectrophotometric methods and the reported method for the analysis of PAR and IBUin pure powder form

Value	RD	PAR			RD	IBU		
		MCR	CC	Reported method^a^ [5]		MCR	CC	Reported method^a^ [5]
Mean	99.95	99.91	99.64	100.25	100.14	100.18	99.97	99.63
SD	0.34	0.65	0.803	0.54	0.81	0.78	0.641	0.65
RSD%	0.340	0.650	0.806	0.538	0.808	0.778	0.641	0.652
n	5	5	5	5	5	5	5	5
Variance	0.115	0.422	0.645	0.291	0.656	0.608	0.411	0.422
Student’s *t* test^(2.306)^	1.036	0.1609	1.412		1.096	0.7081	0.834	
F-value^(6.388)^	2.136	1.690	2.216		1.417	5.382	1.027	

The values in the parenthesis are the corresponding theoretical values of *t* and *F* at *P* = 0.05

^a^Spectrophotometric method using derivative of the ratio spectra method

## Conclusion

Three validated, simple and sensitive spectrophotometric methods were developed for simultaneous determination of PAR and IBU in pharmaceutical preparation without prior separation. The developed methods are simpler, more sensitive than previously published spectrophotometric methods as they did use neither derivative nor multiple manipulating steps; therefore signal-to-noise ratio was improved. The proposed methods could be successfully applied for the simultaneous routine analysis of the combination of PAR and IBU in quality control laboratories.

## Data Availability

All data is included in the manuscript.

## Abbreviations

PAR: Paracetamol
IBU: Ibuprofen
RD: ratio difference spectrophotometric method
MCR: mean centering of ratio spectra method
